# Using health management information system data: case study and verification of institutional deliveries in Ethiopia

**DOI:** 10.1136/bmjgh-2021-006216

**Published:** 2021-08-23

**Authors:** Catherine Arsenault, Bereket Yakob, Munir Kassa, Girmaye Dinsa, Stéphane Verguet

**Affiliations:** 1 Department of Global Health and Population, Harvard T.H. Chan School of Public Health, Boston, Massachusetts, USA; 2 Fenot project, School of Population and Public Health, University of British Columbia, Addis Ababa, Ethiopia; 3 Minister's Office, Ministry of Health, Addis Ababa, Ethiopia; 4 Department of Public Health and Health Policy, Haramaya University, Hararamaya, Ethiopia

**Keywords:** health services research, health systems, health systems evaluation, maternal health

## Abstract

Health management information systems (HMIS) are a crucial source of timely health statistics and have the potential to improve reporting in low-income countries. However, concerns about data quality have hampered their widespread adoption in research and policy decisions. This article presents results from a data verification study undertaken to gain insights into the quality of HMIS data in Ethiopia. We also provide recommendations for working with HMIS data for research and policy translation. We linked the HMIS to the 2016 Emergency Obstetric and Newborn Care Assessment, a national census of all health facilities that provided maternal and newborn health services in Ethiopia. We compared the number of visits for deliveries and caesarean sections (C-sections) reported in the HMIS in 2015 (January–December) to those found in source documents (paper-based labour and delivery and operating theatre registers) in 2425 facilities across Ethiopia. We found that two-thirds of facilities had ‘good’ HMIS reporting for deliveries (defined as reporting within 10% of source documents) and half had ‘very good’ reporting (within 5% of source documents). Results were similar for reporting on C-section deliveries. We found that good reporting was more common in urban areas (OR: 1.30, 95% CI 1.06 to 1.59), public facilities (OR: 2.95, 95% CI 1.38 to 6.29) and in hospitals compared with health centres (OR: 1.71, 95% CI 1.13 to 2.61). Facilities in the Somali and Afar regions had the lowest odds of good reporting compared with Addis Ababa and were more likely to over-report deliveries in the HMIS. Further work remains to address remaining discrepancies in the Ethiopian HMIS. Nonetheless, our findings corroborate previous data verification exercises in Ethiopia and support greater use and uptake of HMIS data for research and policy decisions (particularly, greater use of HMIS data elements (eg, absolute number of services provided each month) rather than coverage indicators). Increased use of these data, combined with feedback mechanisms, is necessary to maintain data quality.

**Summary box**

- Using data from source documents (paper-based registries) in 2425 facilities in Ethiopia, we found that 66% of facilities had good health management information systems (HMIS) reporting for deliveries (defined as reporting within 10% of source documents), and half had very good reporting (within 5% of source documents).
- Hospitals, public facilities and those in urban areas had higher odds of good reporting; facilities in the Somali and Afar regions had the most over-reporting.
- Based on these findings and those of other verification exercises, we argue for greater use of HMIS data elements (eg, absolute number of services provided each month) for research and policy decisions.
- Increased use of these data, combined with feedback mechanisms, is necessary to improve and maintain data quality; country-led HMIS should be the primary source of information for tracking health system performance in low-income and middle-income countries.

## Introduction

Health management information systems (HMIS), based on data reported by health facilities, are an important source of health statistics in low-income and middle-income countries (LMICs). Well-functioning HMIS can provide near real-time data on the performance of health systems and can be used for planning and resource allocation and to track progress on universal health coverage and the Sustainable Development Goals.^1^

The central role of HMIS in improving reporting in LMICs is increasingly recognised.^2^ However, concerns about data quality have hampered the widespread use of HMIS for research and policy decisions across LMICs. In Ethiopia, the Health Sector Transformation Plan (2015–2020) called for a ‘data revolution’ in the way that health data are collected, analysed and disseminated for decision-making.^3^ Since then, several initiatives have aimed to improve the quality of the Ethiopian HMIS.^4–7^ In 2018, Ethiopia adopted the District Health Information Software 2 (DHIS2), a web-based HMIS platform used in over 70 LMICs, and launched a nationwide campaign to improve internet connectivity in health facilities.

There have been few validations of HMIS data in LMICs.^5–12^ Many of these studies have focused on assessing whether intervention coverage indicators estimated using HMIS are consistent with those from household surveys (eg, the demographic and health survey (DHS)).^5 9 11 13^ Intervention coverage indicators (eg, the proportion of women giving birth in health facilities or the proportion of fully immunised children) require two valid data elements: valid numerators (eg, number of women giving birth in facilities) and valid denominators (eg, total number of women giving birth in a catchment area). In addition to estimating intervention coverage indicators, the HMIS should be used to track individual data elements such as the absolute number of services delivered, the number of consultations taking place, and headcounts, cases and deaths from particular conditions. Monitoring the absolute volume of services provided each month can be used for evaluating the effect of new policies aimed at increasing healthcare use or for identifying potential disruptions in health services caused by health system shocks such as COVID-19.^14^

In Ethiopian health facilities, the number of health services provided on a daily basis is first recorded in paper-based registers (tallies or logbooks) in each department (eg, labour and delivery ward register, operating theatre register, safe abortion register, family planning register, newborn unit register, etc).^15^ Data from these paper-based documents are then compiled by a facility statistician (or HMIS focal person) and entered into a digital format for reporting (on a monthly basis for the majority of data). The HMIS focal person is responsible for conducting random accuracy checks and trained in data quality assessments.^16^ It is unclear whether these quality assessments are implemented consistently.^15^

Another way to assess the accuracy of HMIS data is to compare values reported in the HMIS to those found in source documents such as paper-based facility registers. However, studies comparing HMIS with source documents generally require in-person visits, tend to be resource-intensive and thus have generally included small facility samples. For example, similar studies in Ethiopia, Nigeria, Rwanda and Tanzania had samples ranging from only 45 to 400 facilities.^6 8–10 17 18^ These small samples have prevented meaningful assessments of facility characteristics linked with good HMIS reporting.

Our article presents results from a verification study undertaken to gain insights into the quality of HMIS data in Ethiopia. We also describe our experience working with these data in the context of the Fenot project (Achieving Excellence in Primary Healthcare in Ethiopia).^19^ Working closely with the Ministry of Health and Regional Health Bureaus across Ethiopia, the Fenot project aims to improve the use of locally available evidence for planning and decision-making and to build technical capacity to interpret and use evidence.

We used data extracted from facility registers in 2425 facilities in Ethiopia to verify the accuracy of HMIS data for two services: visits for deliveries and caesarean sections (C-sections). We describe the magnitude of agreement between HMIS and source documents, and given our large facility sample, we also highlight some factors associated with good reporting. Finally, we describe our experience working with these data for research and policy translation. Our findings and recommendations may guide future data quality improvement efforts across LMICs.

## Using an existing survey to verify the accuracy of HMIS data

We used a survey conducted in 2016 by the Ethiopian Public Health Institute (EPHI) with support from the Averting Maternal Death and Disability Programme at Columbia University: the Ethiopian Emergency Obstetric and Newborn Care (EmONC) assessment.^15^

The 2016 EmONC assessment was a national cross-sectional census of public and private health facilities that provided maternal and newborn health services in Ethiopia. It included information on infrastructure, human resources, facility service statistics and provider knowledge among others. The survey was conducted from April to December 2016. Data collectors identified labour and delivery ward and operating theatre paper-based registers and extracted the number of deliveries and C-sections performed for the 12 months of January–December 2015. A labour and delivery register was available in all hospitals and maternal and child health (MCH) specialty centres, and in 99% of health centres and clinics. The EmONC assessment included data from 3804 facilities in Ethiopia that provided maternal and newborn health services.

The numbers of deliveries and C-sections reported in the HMIS were compiled for the same months (January–December 2015). At the time of the survey, Ethiopia used an electronic health management information systems (eHMIS) developed by John Snow. The DHIS2 was later adopted in 2018. Data from the eHMIS used in this study were accessed through the Ethiopian Health Data Analytics Platform.^20^ The number of births attended by health personnel was reported each month by all health facilities conducting deliveries in Ethiopia including health centres, primary hospitals, general hospitals, referral hospitals and private health facilities. C-sections were also reported monthly to the HMIS by all facilities with surgical capacity.

Because the two datasets did not contain common health facility identifiers or reliable geocodes (at the time of the survey), we created a statistical code to link health facilities in the EmONC assessment to the HMIS database using region, zone, woreda (ie, district), facility types and facility names. Merging was also checked manually and validated by local researchers. The dataset did not contain any patient-level or identifiable information. The Harvard Longwood Campus Institutional Review Board considers this study exempt from full review as it was based on anonymous, non-identifiable secondary data that did not involve human subjects.

Of the 3804 facilities included in the survey, 2479 (65.2%) were successfully linked to the HMIS. However, labour and delivery registers were missing in 22 facilities and an additional 32 reported no deliveries for the year, leading to a sample of 2425 facilities. The majority of facilities were public health centres, and slightly more than half were in rural areas (table 1). The facilities had an average of 6.2 maternity beds and 26.5 total staff, and 72.3% were in Oromia or in the Southern Nations, Nationalities, and Peoples' Region (SNNP). Our analytical sample was similar to the full census in terms of facility characteristics but tended to include more facilities from certain regions (table 1). For example, Dire Dawa, Oromia and Addis Ababa regions were over-represented, while less than 40% of Beninshangul-Gumuz, Tigray, Amhara, Gambella and Afar facilities were included. Reasons for these regions being under-represented may include important differences in facility names between the HMIS and the survey or that more facilities did not report at all to the HMIS in these regions.

**Table 1:** Characteristics of facilities included in the study compared with the total facilities providing maternal and newborn health services in 2015 in Ethiopia

Facility types	Study sample		All facilities*	
	N	%	N	%
Hospitals†	232	9.6	293	7.7
Health centres	2171	89.5	3459	90.9
MCH specialised centres	22	0.9	52	1.37
Ownership				
Public	2350	96.9	3662	96.3
Private	75	3.1	142	3.7
Location				
Rural	1385	57.1	2307	60.7
Urban	1040	42.9	1497	39.4
Infrastructure and staffing	Median	IQR	Median	IQR
Number of maternity beds	4	3	4	3
Total staff‡	14	10	14	9
Regions	N	%	N	%
Addis Ababa	129	5.3	151	4.0
Afar	30	1.2	77	2.0
Amhara	295	12.2	876	23.0
Benishangul-Gumuz	11	0.5	43	1.1
Dire Dawa	19	0.8	21	0.6
Gambella	10	0.4	27	0.7
Harari	8	0.3	15	0.4
Oromia	1267	52.3	1405	36.9
SNNPR	486	20.0	773	20.3
Somali	94	3.9	161	4.2
Tigray	76	3.1	255	6.7
Total	2425		3804	

*Census of all facilities that provided maternal and newborn health services in 2015 in Ethiopia. Surveyed by the 2016 Ethiopian Emergency Obstetric and Newborn Care assessment.

†Hospital types include referral, general and primary hospitals. MCH specialised centres also include higher clinics.

‡Total staff includes all health staff currently employed by the facility (medical doctors, obstetricians, gynecologists, pediatricians, neonatologists, emergency surgical officer, midwives, nurses, health officers, anesthesiologists, laboratory technicians, pharmacists and health information technologists).

MCH, maternal and child health; SNNPR, Southern Nations, Nationalities, and Peoples' Region.

In 2015, these 2425 facilities reported a total of 1 516 368 million deliveries to the HMIS, and 1 423 723 million deliveries were found in their registers (table 2). At the national level, this represented a 6.5% over-reporting in the HMIS. In contrast, 71 542 C-sections were reported in the HMIS, but 74 061 were found in operating theatre registers, an under-reporting of −3.4% at the national level. Somali and Afar had the highest over-reporting, whereby 47.0% and 34.3% more deliveries were reported to the HMIS compared with those found in registers, respectively. Somali also over-reported C-sections by 27.3%. Addis Ababa and Tigray under-reported both services (table 2).

**Table 2:** Total deliveries and C-sections reported in the Ethiopian HMIS compared with those reported in facility registers in 2425 facilities, by region, in 2015

	Deliveries			C-sections		
	HMIS	Registers	% difference	HMIS	Registers	% difference
Addis Ababa	89 962	94 562	−4.9%	23 133	25 076	−7.8%
Afar	5098	3795	34.3%	130	129	0.8%
Amhara	167 610	151 628	10.5%	9754	10 318	−5.5%
Benishangul-Gumuz	5842	5764	1.4%	714	700	2.0%
Dire Dawa	8988	8184	9.8%	1336	1299	2.9%
Gambella	2620	2435	7.6%	120	135	−11.1%
Harari	5662	5301	6.8%	930	908	2.4%
Oromia	841 731	796 264	5.7%	19 212	18 917	1.6%
SNNPR	311 603	284 023	9.7%	10 976	10 835	1.3%
Somali	23 726	16 138	47.0%	621	488	27.3%
Tigray	53 526	55 629	−3.8%	4616	5256	−12.2%
National	1 516 368	1 423 723	6.5%	71 542	74 061	−3.4%

C-section, caesarean section; HMIS, health management information systems; SNNPR, Southern Nations, Nationalities, and Peoples' Region.

## Verification factors

We created a VF at the facility level calculated as the number of deliveries (or C-sections) reported in the HMIS divided by the number of deliveries (or C-section) found in registers for the year. The resulting ratio was equal to 1 when totals in HMIS and registers were identical, greater than 1 when HMIS over-reported and below 1 when HMIS under-reported (The WHO’s Data Quality Review toolkit calculates a VF by dividing the recounted number of events from source documents by the reported number from the HMIS (ie, registers/HMIS).^13^ For ease of interpretation, we opted to calculate the VF as HMIS divided by registers. With this method, VFs greater than 1 imply HMIS over-reporting, while VFs smaller than 1 imply under-reporting in the HMIS).

**Figure 1:**
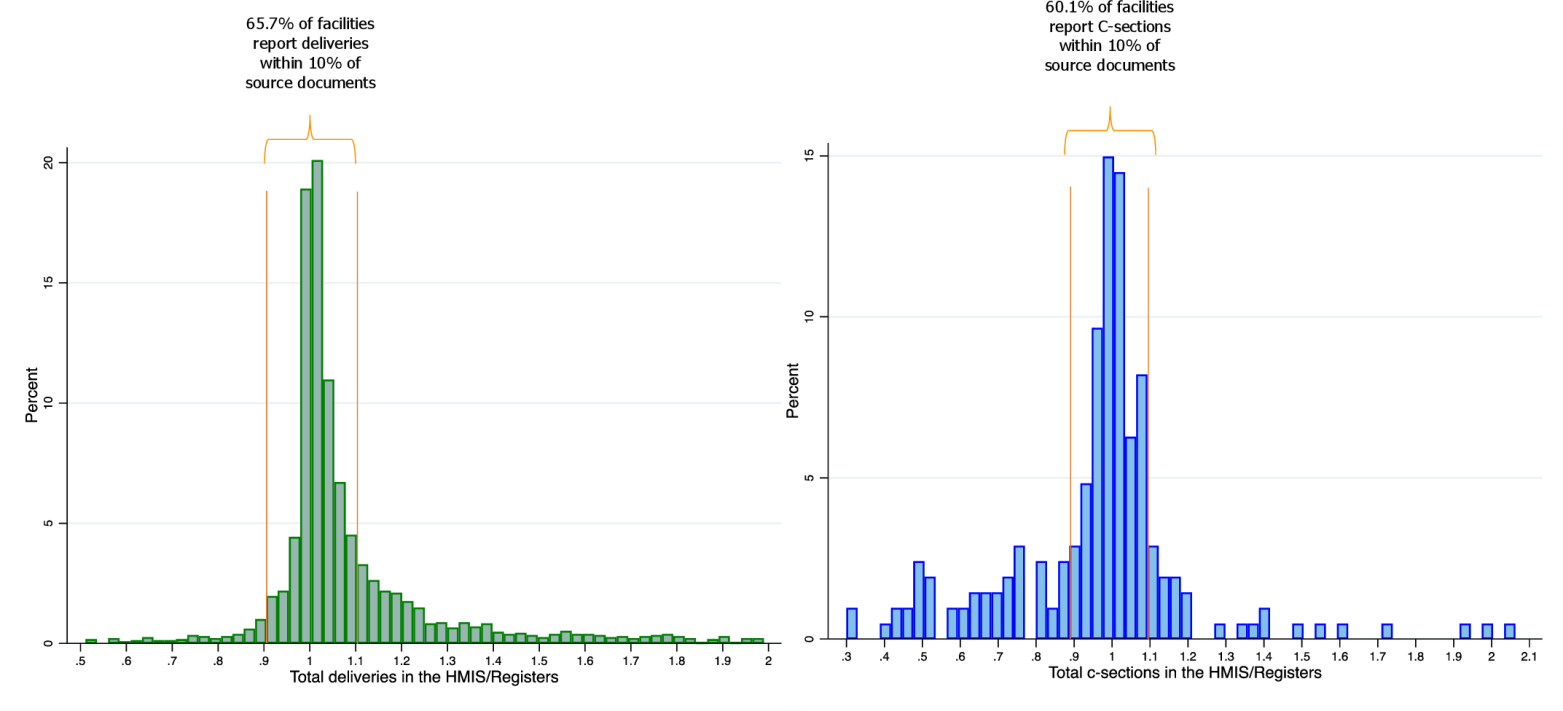
Ratios of deliveries and C-sections reported in the Ethiopian HMIS to those reported in paper-based facility registers (verification factors) by facilities in 2015. HMIS, health management information systems; C-section, Caesarean section. Ratios of the total deliveries and C-sections reported in the HMIS from January to December 2015 to that extracted from health facility registers for the same months (verification factors (VFs)). A VF of 1 means that the number of deliveries in the HMIS matches that found in registers. A VF > 1 indicates a greater number reported in the HMIS, and a VF < 1 indicates a smaller number reported in the HMIS compared to registers. Histogram on the left is truncated at 0.5 and 2.0 to show the region of the plot with most facilities (147 out of 2,425 facilities (6%) are excluded). The plot on the right excludes one facility with a VF of 4 for C-sections.

The VFs for deliveries (HMIS/registers) ranged from 0.05 (indicating that the number of deliveries in the HMIS was only 5% of that found in the registers) to 30.89 (indicating that the number of deliveries in the HMIS was 30.89 times greater than that found in registers), with a mean of 1.26 and a median of 1.03 (figure 1). Across the 208 health facilities that performed C-sections, the VF ranged from 0.30 to 4.00, with a mean of 0.98 and a median of 1.00.

We also created two binary indicators based on the VF for good and very good reporting. Facilities were categorised as having ‘good reporting’ if the HMIS was within 10% of source documents (VF ranges from 0.90 to 1.10). ‘Very good reporting’ included facilities that reported within 5% of source documents (VF ranges from 0.95 to 1.05, a narrower criterion). For deliveries, we found that two-thirds of facilities (65.7%) had good HMIS reporting (VF ranges from 0.90 to 1.10) (figure 1). Among the remaining 34.3% of facilities, 5.0% under-reported and 29.3% over-reported by more than 10%. Half of facilities (50.5%) had very good HMIS reporting (VF ranges from 0.95 to 1.05). For C-sections, 60.1% of facilities had good HMIS reporting and 42.8% had very good reporting.

**Figure 2:**
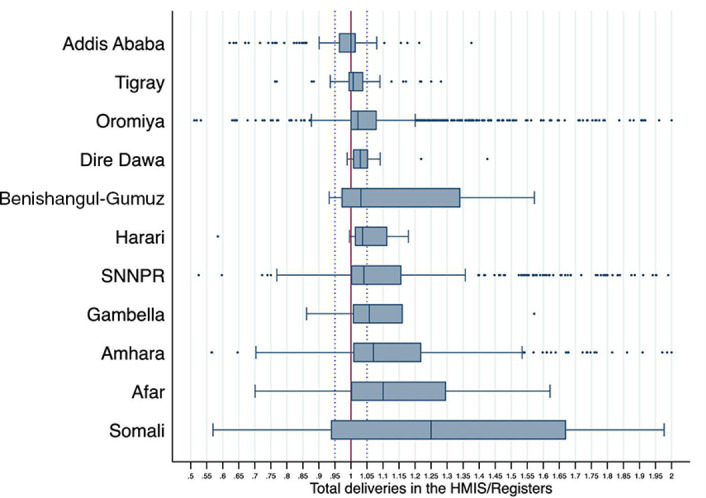
Verification factors (total deliveries in HMIS divided by that in registers) by Ethiopian region in 2015. HMIS, health management information systems; SNNPR, Southern Nations, Nationalities, and Peoples’ Region. Boxplots are truncated at 0.5 and 2.0 to show the region of the plot with most facilities (147 of 2,425 facilities (6%) are excluded). Regions are sorted by median VF after truncation. The red line at 1 indicates perfect agreement. The blue dotted lines show the region of “very good” reporting: HMIS is within 5% of registers. The median VF in Benishangul-Gumuz was 1.17 before truncation.

In figure 2, we summarised the VFs for deliveries by region. Median VFs were within 5% of source documents (very good reporting) in six regions and greater than 1.05 in five regions. Somali and Afar, in particular, had the most over-reporting.

## Facility characteristics associated with good reporting

To explore potential factors associated with good reporting we used multivariable logistic regression models with robust standard errors, regressing the binary indicator for good reporting (reporting within 10% of source documents) on a series of facility characteristics: facility type (hospital, health centre and MCH specialised centre), ownership (public vs private), location (urban vs rural), and region fixed effects. We repeated the regression using the indicator for very good reporting (reporting within 5% of source documents). We removed outliers by excluding the top and bottom 1% of facilities based on the VF estimates.^21^ The analysis thus included 2376 facilities with VFs ranging from 0.47 to 6.02.

We found that the odds of good reporting were higher in urban compared with rural areas (OR: 1.30, 95% CI 1.06 to 1.59), in public compared with private facilities (OR: 2.95, 95% CI 1.38 to 6.29) and in hospitals compared with health centres (OR: 1.71, 95% CI 1.13 to 2.61) (table 3). Compared with Addis Ababa (the capital region), nearly all regions had lower odds of good and very good reporting.

**Table 3:** Odds ratios for the associations between facility characteristics and ‘good’ and ‘very good’ HMIS reporting (HMIS and paper-based registers are within 10% and 5%, respectively)

	N	Good reporting (VF ranges from 0.90 to 1.10)				Very good reporting (VF ranges from 0.95 to 1.05			
		OR	95% CI		P value	OR	95% CI		P value
Facility type* (ref. health centres)									
Hospitals	222	1.71	1.13	2.61	0.012	1.41	0.98	2.02	0.061
MCH specialised centres	21	0.80	0.25	2.57	0.707	0.58	0.18	1.84	0.358
**Public ownership (ref. private)**	2307	2.95	1.38	6.29	0.005	2.64	1.30	5.38	0.007
**Urban location (ref. rural)**	1018	1.30	1.06	1.59	0.012	1.40	1.16	1.69	0.000
**Regions (ref. Addis Ababa)**									
Afar	24	0.09	0.03	0.24	0.000	0.09	0.03	0.25	0.000
Amhara	292	0.20	0.11	0.36	0.000	0.23	0.14	0.38	0.000
Benishangul-Gumuz	11	0.15	0.04	0.51	0.002	0.18	0.05	0.66	0.010
Dire Dawa	19	0.74	0.22	2.46	0.617	0.59	0.22	1.61	0.303
Gambella	9	0.16	0.04	0.63	0.009	0.05	0.01	0.39	0.004
Harari	8	0.27	0.05	1.40	0.119	0.30	0.07	1.39	0.123
Oromia	1258	0.64	0.38	1.10	0.107	0.53	0.33	0.85	0.009
SNNPR	478	0.34	0.20	0.59	0.000	0.31	0.19	0.51	0.000
Somali	77	0.03	0.01	0.08	0.000	0.01	0.00	0.05	0.000
Tigray	76	1.06	0.46	2.43	0.884	1.07	0.53	2.17	0.842

*Hospital types include referral, general and primary hospitals. MCH specialised centres also include higher clinics.

HMIS, health management information systems; MCH, maternal and child health; ref., reference; SNNPR, Southern Nations, Nationalities, and Peoples' Region; VF, verification factor.

## Challenges and recommendations

### Data elements from HMIS should be used more broadly for policy and research

Other studies have verified HMIS data elements in Ethiopia using source documents. In 2016, the EPHI and the Federal Ministry of Health (FMOH), with technical assistance from the WHO, conducted a data verification study using source documents from approximately 400 facilities, including all hospitals and a representative sample of health centres and private clinics.^8^ The verification covered seven HMIS data elements reported over 3 months (July–September 2015). The study found mean VFs (defined as source documents divided by HMIS) of 0.92 for antenatal care visits, 1.01 for deliveries, 0.96 for the third dose of pentavalent vaccine, 0.95 for prevention of mother-to-child transmission of HIV, 0.95 for tuberculosis, 0.92 for malaria and 0.80 for family planning. This indicates that, for the exception of family planning, all indicators were reported within 10% of source documents on average. Endriyas and colleagues conducted a verification in 163 facilities in the SNNPR.^6^ They found that the proportion of facilities reporting accurately (within 10% of source documents) ranged from 46.6% for the number of fourth antenatal care visits to 84.7% for deliveries and 96.9% for maternal deaths.^6^

Data elements in the HMIS include the absolute numbers of services delivered, of consultations taking place, and headcounts, cases and deaths from particular conditions. Although some work remains to perfect reporting, we believe that our findings and those of the studies cited previously support greater use and uptake of HMIS data elements for research and policy decisions in Ethiopia.^6 8^ Furthermore, these studies were conducted using data from 2015 to 2017. HMIS quality has likely improved since, given the multitude of training initiatives and interventions to improve data quality that have recently taken place in Ethiopia.^5 22–24^ The expansion of internet connectivity along with the adoption of DHIS2 in 2018 may have facilitated standardisation in reporting and further improved data quality in Ethiopia.

### Data quality improvement should target the private sector, smaller facilities and those in rural areas

Reasons for inconsistent HMIS reporting in LMICs remain unclear and likely differ across facility types, indicator types, regions and countries. In our study, private facilities had poorer reporting and should be prioritised for HMIS data quality interventions. Lack of any HMIS reporting or poor-quality reporting by the private sector has been found in other countries.^18 25 26^ There is an urgent need to develop incentives for private facilities to report more accurately to national HMIS.

Hospitals also had better quality reporting than health centres.^15^ Overall, the number of C-sections tended to be better reported than routine deliveries, likely because they are conducted primarily in hospitals. Hospitals are generally better equipped and employ more staff for record keeping and data reporting.^27^ The EmONC assessment found that only 66% of health centres in Ethiopia had a dedicated person responsible for maternal and newborn health data reporting (such as a data manager or health information technologist) compared with 90%–100% in hospitals.^15^ As a subanalysis, we regressed the binary indicators for good and very good reporting on urban area, presence of a designated data manager and region fixed effects for health centres only (N=2103). We found that the odds of good and very good reporting were 1.55 and 1.24 times higher, respectively, in health centres with a dedicated data manager (data not shown). Ensuring that each facility has a dedicated staff for managing and reporting health service data would be a valuable strategy to improve HMIS accuracy.

Facilities in Somali and Afar had the lowest odds of good reporting and were substantially more likely to over-report in the HMIS (table 2 and figure 2). These two regions are predominantly rural and inhabited by pastoral communities. Though it is unclear why they tend to over-report, facilities in these rural areas face structural challenges that may affect HMIS reporting, including lack of reliable electricity or computers. Basic infrastructure is necessary for HMIS reporting in a digital format.

### Population coverage indicators from HMIS may not be reliable yet

Other studies have compared population coverage indicators between the Ethiopian HMIS and DHS and found very low agreement. Adane and colleagues estimated that the proportion of fully vaccinated children in Ethiopia would be 89% in the HMIS but only 39% in the DHS.^5^ The Ethiopian FMOH also routinely compares health intervention coverage using the HMIS to those from the DHS.^28^ Coverage estimates from HMIS often differ from household survey estimates, and it is not uncommon to find coverage estimates that are greater than 100%. Coverage indicators (reported as proportions) require both valid numerators (eg, number of fully vaccinated children aged 1 year) and denominators (total number of 1-year-old children). Discrepancies in coverage measures between HMIS and population-based surveys are the product of both inaccurate service volumes (numerators) and inaccurate target populations (denominators). Target populations are estimated using national census data on population age and sex, which are projected using assumptions about fertility, mortality and migration. In Ethiopia, the latest population census was conducted in 2007.^29^ A 2018 study across 14 Eastern and Southern African countries found that the median year of the most recent census used for population projections was 2009.^11^ The same study also found inaccuracies in population projection calculations and inappropriate use of population growth rates and crude birth rates.^11^ Without accurate population projections, the HMIS may not produce accurate coverage estimates.

## Strengths and limitations of this study

Our study is one of a few to verify the accuracy of HMIS data elements and provides a framework and methods for others to assess HMIS data quality. Unlike other data quality studies, we were able to include a large number of facilities (N=2425), which allowed us to investigate the facility characteristics associated with good reporting. Nonetheless, our study presents several limitations. First, we used data from paper-based registers as the gold standard to verify HMIS data. These source documents may also be incomplete or may contain errors. Nonetheless, our study simply relied on tallying the number of deliveries and C-sections performed and did not include detailed patient information from source documents. Second, our analysis includes only two data elements from the HMIS (deliveries and C-sections). The Ethiopian HMIS contains over 100 data elements and indicators on volumes of services (including reproductive, maternal, newborn and child health services, vaccinations, tuberculosis care, HIV care and chronic disease care). It also includes quality-of-care indicators (eg, the proportion of asphyxiated neonates who were resuscitated and survived, and the proportion of hypertensive patients with controlled blood pressure) and institutional deaths (eg, maternal, neonatal and inpatient deaths). The precision of other indicators may differ, especially those with more complex definitions or that are more complex to measure. Nonetheless, other studies have found similar reporting quality for other HMIS data elements in Ethiopia.^6 8^ Third, our study includes only 64% of all facilities that provided maternal and newborn care in Ethiopia at the time. The other 36% could not be linked between the two datasets, likely due to differences in facility or woreda names or because they did not report to the national HMIS. Though our sample was largely consistent with the facility census in terms of facility characteristics, certain regions were over-represented. For example, we were able to link 85% and 90% of all facilities in Addis Ababa and Dire Dawa, but only 58% and 39% of all facilities in Somali and Afar. Our results may therefore slightly overestimate data quality at the national level. Finally, the EmONC assessment did not include health posts. Health posts, staffed with community health workers, are the lowest levels of the primary healthcare system. Although health posts are not supposed to conduct deliveries, it is possible that some women gave birth at these facilities.

## Conclusions

Across a large sample of health facilities in Ethiopia, we found that two-thirds of facilities had good HMIS reporting for institutional deliveries (defined as reporting within 10% of source documents for the year). Urban facilities, hospitals and public facilities had higher odds of good reporting. The Somali and Afar regions had the most over-reporting. Despite the limitations stated earlier, our results and those of similar verification studies support greater use and uptake of HMIS data for research and policy decisions in Ethiopia. Increased use of these data, combined with feedback mechanisms, is necessary to improve and maintain data quality. Rather than relying heavily on externally driven surveys conducted once every 5 years, Ethiopia and countries with similar HMIS must invest in building local capacity for reporting and analysing HMIS data. The adoption of the DHIS2, a web-based standardised HMIS platform, across 73 LMICs offers a renewed opportunity for global efforts to contribute to improving these data systems.^10 11 30^

Future research should further explore and address the underlying causes of poor HMIS reporting in certain areas. Efforts to improve HMIS should also consider reviewing the total number of data elements required for reporting by facilities every month, the clarity of reporting guidance and definitions, and the estimation of target population size used by the HMIS to estimate coverage indicators. Moving forward, we conclude that country-led HMIS should be the primary source of information for tracking health system performance in LMICs.

## Data Availability

Data may be obtained from a third party and are not publicly available.
